# The green synthesis of gold nanoparticle using extract of *Virola oleifera*

**DOI:** 10.1186/1753-6561-8-S4-P29

**Published:** 2014-10-01

**Authors:** Bárbara Milaneze, Wanderson Keijok, Oliveira Jairo, Peruch Brunelli, Bartochevis Janine, Liqui Larissa, Prado Adilson, Endringer Denise, Pontes Maria, Ribeiro Moises, Nogueira Breno, Guimarães Marco

**Affiliations:** 1 Universidade Federal do Espírito Santo, Vitória, Brazil; 2 Universidade de Vila Velha Instituto Federal do Espírito Santo, Vitoria, Brasil

## Background

The green synthesis consists of an environmentally friendly method of producing gold nanoparticles (AuNP's). Physical and chemical syntheses have energy intensive and may involve toxic chemicals, while biological techniques are cost-effective, clean, non-toxic and environmentally appropriate.

*Virola oleifera *is widely used in folk medicine. The bark of the trunk when scraped produce a resin rich in phenolic compounds, which is used against bleeding hemorrhoids, cramping, and also has healing action of chronic wounds and ulcers, diarrhea and counter hemoptysis. It is known that the presence of phenolic extract gives the reducing action, but studies need to be done to understand what substances are involved and what the mechanisms of formation of newly synthesized nanoparticles.

Thus, the aim of this study was to describe a new route for the synthesis of AuNP's using resin of *Virola oleifera *with future application in nanoscience from the synthesis of nanoparticles for applications as nano biosensors.

## Methods

To check the effect of variables on conversion of the reaction, as well as finding the conditions that maximized the synthesis of nanoparticles, one factorial design (3²) with 3 levels and 2 variables was done. The concentration of the reducing agent (0.5, 1.0 and 2.0 mL), and the synthesis time (5, 10 and 15 min). These intervals were defined based on the literature.

To prepare nanoparticles, was used the gold precursor solution (HAuCl_4 _with 2,5x10^-4 ^M) and as reducer agent the resin lyophilized of *Virola oleifera *(1mg/mL), both dillueted in distilled water. Based on the experimental design, the solution of the reducing agent was added to the gold solution and stirred for predefined times.

AuNP's samples were collected after the synthesis step and had their optical properties assessed by spectrophotometry UV-visible (SHIMADZU). The size and morphology of AuNP's were examined by transmission electron microscopy (JEM-1400, JEOL Inc, USA).

## Results and conclusions

The absorption spectra of UV-Vis showing that the synthesis resin of *Virola oleifera *leads to the formation of nanoparticles with different optical properties according to the synthesis time and concentration of reducing agent.

The results of the electronic spectra of the solutions obtained and the analysis in the transmission electron microscope showed the difference in the absorbance spectrum of the particles. It was observed that the concentration of the reducing agent was significant in the synthesis process, and that the absorbance peaks were found in the highest concentrations of reducing agent, with consequent increase in nanoparticle size.
